# A Double-Edged Algorithm Attitude: How Appreciation and Aversion Shape Students’ AI Learning Anxiety in Higher Education

**DOI:** 10.3390/bs16060932

**Published:** 2026-06-05

**Authors:** Zhaolin Lu, Jiayuan Guo, Tian Yuan, Yue Zhang, Jiajie Yang, Yuxuan Du, Minghua Chen, Mingyi Xie, Liangyu Xian, Hui Cao, Kexin Zhang

**Affiliations:** 1 School of Design and Arts, Beijing Institute of Technology, No. 5 South Street, Zhongguancun, Haidian District, Beijing 100081, China; zhaolin.lu@bit.edu.cn (Z.L.); jiayuan.guo@bit.edu.cn (J.G.); 2 School of Architecture and Art, Hefei University of Technology, No. 193 Tunxi Road, Hefei 230009, China

**Keywords:** artificial intelligence, higher education, learning anxiety, algorithm attitude, structural equation modeling, artificial neural network

## Abstract

Artificial intelligence is rapidly entering higher education, yet many students experience anxiety when learning to use it. This study examines how performance expectations, perceived explainability, and perceived ethical risks shape two algorithm attitudes, algorithm aversion and algorithm appreciation, and how these attitudes influence artificial intelligence learning anxiety. Using a hybrid partial least squares structural equation modeling–artificial neural network (PLS-SEM–ANN) approach, this study analyzed survey data from 409 university students. Results show that both algorithm aversion and algorithm appreciation significantly increase artificial intelligence learning anxiety, although the effect of algorithm aversion is much stronger, supporting an approach–avoidance account. Perceived ethical risk is the strongest predictor of algorithm aversion but has no significant effect on algorithm appreciation. By contrast, performance expectations and perceived explainability strengthen algorithm appreciation while also showing weaker positive effects on algorithm aversion. These findings suggest that, in educational settings, stronger performance value and greater explainability do not simply reassure students; they can also increase pressure by making errors, responsibility, and the need to use artificial intelligence effectively more salient. The artificial neural network results corroborate these patterns. This study extends research on algorithm attitudes and offers guidance for creating more supportive artificial intelligence learning environments.

## 1. Introduction

With the rapid development of generative artificial intelligence (AI), tools such as ChatGPT (GPT-3.5) and DeepSeek-R1 have quickly entered higher education and are increasingly embedded in students’ information retrieval, writing support, problem solving, and learning assistance (Cai et al., 2025). Compared with earlier educational technologies, generative AI not only improves learning efficiency and task performance but also reshapes how students understand knowledge acquisition, skill development, and the learning process (Y. Zhu et al., 2025). As a result, many universities have begun to integrate AI into teaching and learning practices and regard it as an important tool for strengthening learning support and developing future-oriented competencies. However, the spread of AI in education does not only produce empowering benefits. It also introduces new forms of uncertainty, accountability pressure, and psychological burden into students’ learning experiences (J. J. H. Kim et al., 2025). In particular, when learning how to use AI and when using AI for academic tasks, some students experience worry, tension, unease, or fear of making mistakes, suggesting that AI learning anxiety has become an issue that deserves attention in higher education (Almaiah et al., 2022).

Although previous studies have examined students’ general responses to AI, the mechanisms underlying AI learning anxiety remain underexplored (Kotlyar & Krasman, 2025; Idroes et al., 2023). Existing research has usually explained students’ negative reactions to AI in terms of perceived difficulty, uncertainty, or risk (Kulal, 2025). Dominant frameworks such as the Technology Acceptance Model and the Unified Theory of Acceptance and Use of Technology mainly address whether students see AI as useful and easy to use, and whether they are willing to adopt it. They are less well suited to explaining the more ambivalent emotional responses that may arise when students engage with AI in educational settings (Venkatesh, 2022). Even when prior studies discuss attitudes toward algorithms, they often treat them as moving in a single direction, either positive or negative (Park & Yoon, 2025). This leaves limited room for understanding algorithm appreciation and algorithm aversion as two attitudes that may coexist, and little is known about how their coexistence may shape AI learning anxiety (Jussupow et al., 2024).

To address this gap, this study argues that students’ psychological responses to AI in higher education do not necessarily follow a simple one-dimensional continuum from acceptance to rejection. On the one hand, students may recognize AI’s efficiency, convenience, and value for learning (Zhou et al., 2024); on the other hand, they may remain cautious or even resistant because of concerns about privacy leakage, bias and unfairness, hallucinated content, ambiguous academic integrity boundaries, and unclear responsibility attribution (Al-Chaer, 2026). This coexistence creates a double-edged algorithm attitude dilemma: students may appreciate AI as a valuable learning support while simultaneously developing aversion toward its risks, uncertainty, and potential consequences. Algorithm appreciation and algorithm aversion may therefore coexist and jointly shape students’ emotional experiences in the process of learning with AI. Introducing this dilemma in the context of AI learning anxiety allows this study to move beyond a one-sided acceptance-or-rejection perspective and examine how positive and negative algorithmic attitudes operate together.

The study develops a research model based on the Stimulus–Organism–Response (S-O-R) framework (Jacoby, 2002; Zhao et al., 2020). It introduces performance expectancy of AI, perceived explainability, and perceived ethical risk as antecedents and examines how they influence students’ AI learning anxiety through algorithm appreciation and algorithm aversion. Methodologically, this study combines partial least squares structural equation modeling (PLS-SEM) and artificial neural networks (ANN) to examine the relationships among variables and the mediating mechanisms, and to identify the relative importance of key predictors. By integrating the S-O-R perspective with a combined analytical approach, this study aims to deepen understanding of the psychological mechanisms underlying AI learning anxiety in higher education and to provide theoretical support and practical guidance for universities seeking to help students engage with AI in a more rational, stable, and less anxious way.

## 2. Literature Review

### 2.1. Algorithm Appreciation

With the widespread diffusion of AI technologies, individuals have increasingly shown positive attitudes toward algorithmic advice in judgment and decision-making tasks (Bogert et al., 2021). When algorithmic and human advice are of equal quality, people often perceive algorithms as superior in information integration and decision objectivity; this tendency has been conceptualized as algorithm appreciation (Logg et al., 2019). Unlike general technology trust, algorithm appreciation emphasizes users’ active recognition of and reliance on algorithmic decision-making capabilities. It reflects the belief that algorithms can produce more objective and accurate judgments through large-scale data processing and pattern recognition (Qin et al., 2025).

Prior research suggests that algorithm appreciation is mainly rooted in perceived performance advantages. Algorithms are often viewed as more stable, scalable, and consistent in processing complex information, and as less vulnerable than humans to emotional fluctuation, cognitive overload, or subjective bias (Hu et al., 2026). In addition, explainability is an important condition shaping positive evaluations of algorithms. When systems provide clear rationales or understandable explanations, users are more likely to comprehend their underlying logic, which in turn enhances trust and reliance (Laato et al., 2022). Algorithm appreciation, therefore, arises not only from perceived technical competence but also from cognitive understanding and perceived control.

In educational settings, algorithm appreciation has become increasingly relevant as AI systems are embedded in adaptive learning, assessment, prediction, and instructional support (Zawacki-Richter et al., 2019). When students perceive AI systems as accurate, timely, and useful, they are more likely to form positive evaluations and rely on algorithmic support in learning-related decisions (da Silva et al., 2024). This tendency is particularly visible in generative AI and learning analytics contexts, where the perceived reliability and efficiency of AI outputs can strengthen students’ acceptance of algorithmic recommendations (Cengiz & Peker, 2025). Similarly, in human–AI collaborative learning environments, students’ reliance on AI feedback reflects their recognition of algorithmic capability and may be regarded as an important manifestation of algorithm appreciation in education (Chassignol et al., 2018).

However, existing research in educational contexts has focused primarily on behavioral outcomes such as technology acceptance and usage intention (C. Chen et al., 2025). Although some studies grounded in the Technology Acceptance Model have examined relationships among AI anxiety, attitudes, and behavioral intention (Kai et al., 2026), relatively little is known about how algorithm appreciation is formed in educational settings or how it shapes students’ emotional learning experiences. A more refined understanding of algorithm appreciation is therefore needed to clarify its psychological role in AI-supported learning.

### 2.2. Algorithm Aversion

Despite the recognized performance advantages of algorithms, individuals do not always respond positively to algorithmic decision-making. In some contexts, they develop distrust of and avoidance toward algorithms, a phenomenon commonly described as algorithm aversion (Dietvorst et al., 2015). For example, in professional tasks that require subjective judgment, individuals may still prefer human judgment even when algorithms demonstrate superior performance, due to considerations related to domain-specific expertise or pre-existing expectations toward human experts (Burton et al., 2020). This suggests that negative attitudes toward algorithms are shaped not only by objective performance but also by subjective perceptions and biases.

Prior research indicates that algorithm aversion can be understood at the levels of the individual, the system, and the context. At the individual level, users tend to be more sensitive to algorithmic errors and less tolerant of algorithmic failure than of human error (Dietvorst et al., 2015). At the system level, the black-box nature of algorithms can reduce perceived control and make it difficult for users to understand or intervene in decision processes, thereby undermining trust (Shin, 2021). At the contextual level, ethical and social risks have become increasingly salient. Concerns about privacy breaches, algorithmic bias, opacity, and unclear responsibility attribution may weaken trust in AI systems and intensify algorithm aversion, especially in high-stakes or risk-sensitive settings (de Jong et al., 2025).

In educational contexts, algorithm aversion is likewise an important factor shaping students’ experiences with AI. As AI becomes more deeply integrated into education, students are not only users of AI systems but also potential subjects of algorithmic evaluation and prediction, which may heighten their caution toward such systems (Kasneci et al., 2023). Previous studies show that students may worry about the fairness of algorithmic assessment, the security of data use, and the possible replacement of teachers by AI, all of which can reinforce resistance to algorithmic intervention (Akgun & Greenhow, 2022). Algorithmic recommendations may also reduce students’ sense of autonomy when learning paths or content are strongly shaped by system logic, thereby triggering resistance (Kizilcec & Lee, 2022). Moreover, the rise of generative AI has added further complexity, as students may value its efficiency while simultaneously fearing its substitutive effects on their own capability development (Rudolph et al., 2023).

Taken together, algorithm aversion in higher education may arise from error sensitivity, limited perceived control, and ethical or capability-related concerns. Importantly, algorithm aversion does not necessarily exclude algorithm appreciation. Students may simultaneously recognize the efficiency and utility of AI while remaining wary of its risks and consequences. This coexistence of positive and negative algorithmic evaluations provides an important basis for understanding AI learning anxiety.

### 2.3. AI Learning Anxiety

As AI becomes increasingly integrated into higher education, growing attention has been paid to students’ emotional experiences when learning and using AI. In educational psychology, learning anxiety generally refers to feelings of tension, unease, and stress arising from insufficient ability, task difficulty, or uncertainty about outcomes (Pekrun, 2006). Related research has examined similar forms of anxiety in technology-mediated contexts, such as computer anxiety, technology anxiety, and online learning anxiety (Saadé & Kira, 2009). Because learning with AI often involves navigating rapidly evolving technologies, evaluating AI-generated outputs, and adapting learning strategies, it may generate distinctive forms of emotional pressure for students (Y.-M. Wang et al., 2024).

In this study, such pressure is conceptualized as AI learning anxiety, that is, the anxiety students experience due to psychological factors when engaging with AI tools in learning contexts (Y. Y. Wang & Wang, 2022). Existing studies suggest that AI learning anxiety is shaped by multiple factors, including perceived uncertainty about AI systems, concerns about losing control over technology (Wen et al., 2024), and perceived inadequacy in coping with emerging technologies (White et al., 2024). C. Chen et al. (2025) further found that AI learning anxiety has a complex relationship with motivated learning, generating psychological burden while at the same time stimulating greater learning effort. Overall, AI learning anxiety has been identified as an important factor influencing students’ acceptance and use of AI technologies, with high levels of anxiety potentially reducing learning efficiency, self-efficacy, and willingness to engage with AI-supported learning (Y.-M. Wang et al., 2024).

However, in educational settings, students’ perceptions and evaluations of AI are often characterized by the coexistence of both positive and negative attitudes (Cengiz & Peker, 2025). This suggests that students’ learning and use of AI may be shaped simultaneously by recognition of AI’s capabilities and value, as well as concerns about its potential risks and limitations (Kai et al., 2026). This more complex evaluative process remains underexplored. Accordingly, this study introduces algorithm appreciation and algorithm aversion as two relatively independent yet coexisting attitudinal dimensions and examines how they jointly shape the formation of AI learning anxiety.

## 3. Research Model and Hypothesis

This study adopts the Stimulus–Organism–Response (S-O-R) framework as the overarching theoretical foundation and model structure. The S-O-R model posits that external environmental stimuli do not directly determine individuals’ final responses; rather, they first influence individuals’ internal psychological states, which subsequently shape emotional and behavioral outcomes (Song et al., 2025).

In the context of AI-supported learning in higher education, students’ perceptions of external characteristics of AI, such as performance, explainability, and ethical risks, constitute key stimulus factors (Bai, 2025). These stimuli further influence students’ internal attitudinal states toward algorithms, including algorithm appreciation and algorithm aversion, which in turn affect their psychological response in the form of AI learning anxiety (Vafaei-Zadeh et al., 2025).

**Figure 1:**
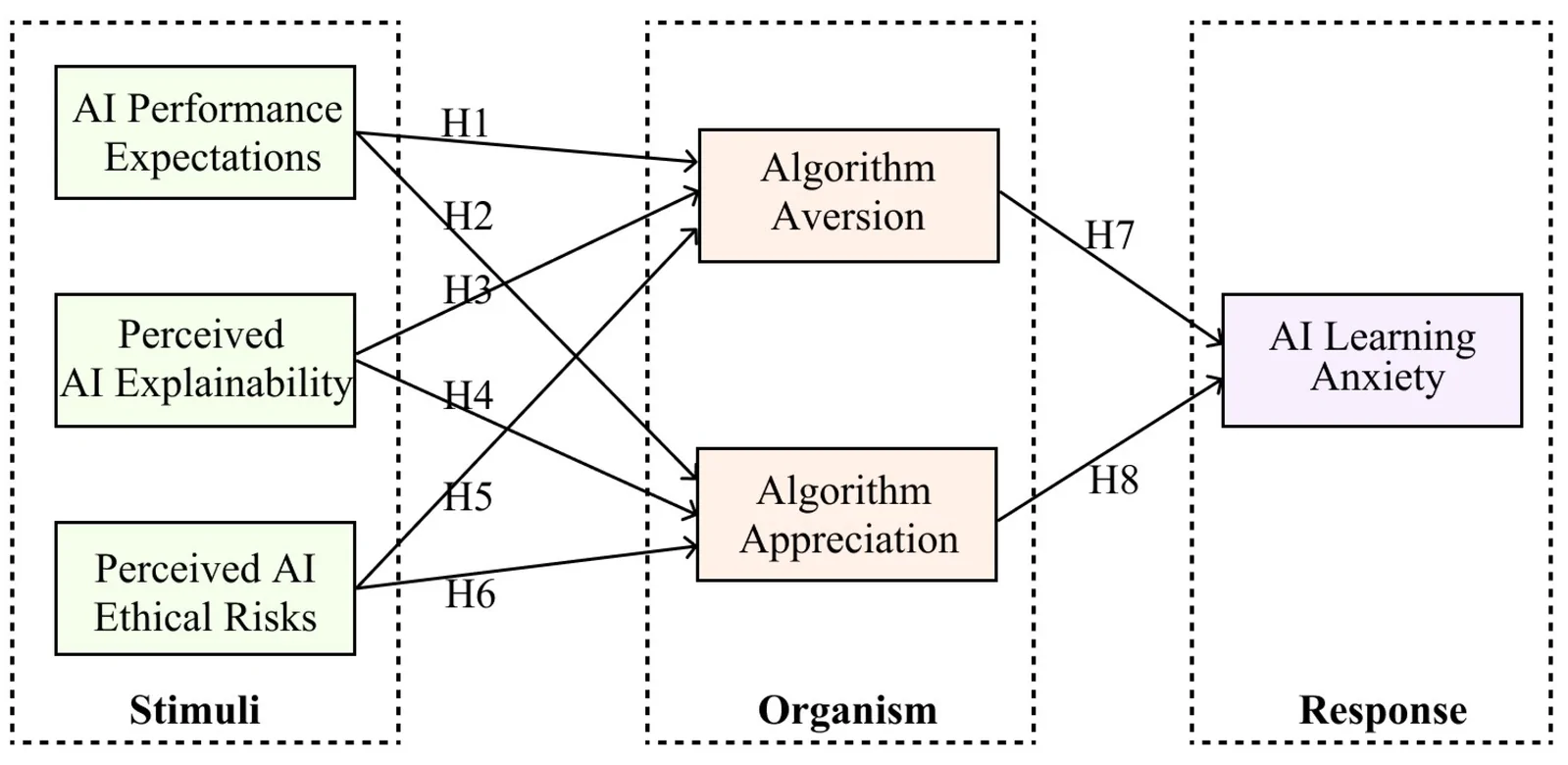
Research model and hypotheses.

Accordingly, this study conceptualizes AI Performance Expectations (APE), perceived AI Explainability (AE), and Perceived AI Ethical Risks (AER) as stimulus-level variables; Algorithm Appreciation (AAP) and Algorithm Aversion (AAV) as organism-level variables; and AI Learning Anxiety (ALA) as the response-level variable. Based on this framework, the research model is constructed, and the following hypotheses are proposed. The hypothetical model is shown in Figure 1.

### 3.1. AI Performance Expectations and Algorithmic Attitudes

As AI becomes increasingly embedded in learning processes, students’ evaluations of AI tools are often shaped first by their perceptions of system performance. AI performance expectations refer to individuals’ overall assessment of the accuracy, effectiveness, and usefulness of AI systems in task execution (Musawa et al., 2024). Prior research has identified perceived performance as an important antecedent of technology attitudes. In the context of learning with generative AI, when students perceive that AI can provide high-quality feedback and effective support, they are more likely to form positive evaluations and rely on its recommendations (Kai et al., 2026).

However, the effect of performance expectations may not be exclusively positive. High expectations regarding AI performance may also heighten users’ sensitivity to system errors. When AI outputs fail to meet expected standards, disappointment and distrust may emerge, thereby fostering more negative evaluations of algorithms (Glikson & Woolley, 2020). Accordingly, AI performance expectations may influence both algorithm appreciation and algorithm aversion.

Based on the above analysis, the following hypotheses are proposed:

**H1.**  *AI performance expectations have a significant effect on algorithm aversion.*

**H2.**  *AI performance expectations have a significant effect on algorithm appreciation.*

### 3.2. Perceived AI Explainability and Algorithmic Attitudes

The black-box nature of AI has long been a central concern in AI development, making explainability a critical factor shaping user attitudes (Hassija et al., 2024). In educational contexts, students are concerned not only with whether AI systems are useful but also with whether their reasoning and decision-making processes are transparent and understandable (Holmes et al., 2019). Perceived AI explainability, therefore, serves as an important link between technological characteristics and users’ psychological responses.

Existing research suggests that explainability can enhance trust in AI systems and thereby improve user acceptance (Cheung & Ho, 2025). In human–AI interaction, explainability may promote reliance on AI by reducing uncertainty and strengthening cognitive understanding (J. Kim et al., 2024). In educational settings, when AI systems provide explanations aligned with learning tasks, both students and teachers are more likely to trust the system and accept its recommendations (Feldman-Maggor et al., 2025). At the same time, prior studies also suggest that the relationship between explainability and trust is not always linear. When explanatory information is overly complex or difficult to interpret, it may increase cognitive load and weaken users’ overall evaluations of the system (Bove et al., 2024). Perceived AI explainability may therefore shape both positive and negative algorithmic attitudes.

Based on the above analysis, the following hypotheses are proposed:

**H3.**  *Perceived AI explainability has a significant effect on algorithm aversion.*

**H4.**  *Perceived AI explainability has a significant effect on algorithm appreciation.*

### 3.3. Perceived AI Ethical Risks and Algorithmic Attitudes

AI ethical risks refer to users’ concerns about potential problems arising from AI use, including privacy breaches, algorithmic bias, unclear responsibility attribution, and technological misuse. Such concerns constitute an important cognitive dimension in AI-related decision-making (Morante et al., 2024). Prior research indicates that algorithms lack moral agency and cannot assume responsibility in the same way as human actors; when responsibility attribution is perceived as ambiguous, users are more likely to develop suspicion and distrust toward algorithms, thereby strengthening algorithm aversion (Yu et al., 2025).

In educational contexts, students’ perceptions of AI ethical issues also shape their attitudes toward AI use. When students believe that AI systems may compromise fairness in evaluation or threaten data privacy, they are more likely to experience resistance and other negative reactions (Holmes et al., 2022). At the same time, perceptions of ethical risk may also influence broader evaluations of AI systems, including judgments of their reliability and appropriateness for use (Peng & Zhao, 2024). Perceived AI ethical risks may therefore not only intensify algorithm aversion but also affect algorithm appreciation.

Based on the above analysis, the following hypotheses are proposed:

**H5.**  *Perceived AI ethical risks have a significant effect on algorithm aversion.*

**H6.**  *Perceived AI ethical risks have a significant effect on algorithm appreciation.*

### 3.4. Algorithmic Attitudes and AI Learning Anxiety

As AI becomes an increasingly important tool in learning environments, students’ attitudinal and emotional responses to AI have attracted growing scholarly attention. Prior research suggests that negative attitudes toward technology are often associated with distrust and anxiety (Venkatesh, 2022). In educational contexts, algorithm aversion may lead students to perceive AI systems as unreliable, threatening, or difficult to trust, thereby fostering avoidance tendencies. Yet because AI is becoming increasingly difficult to avoid in contemporary learning settings, such resistance may accumulate into sustained psychological pressure and further intensify AI learning anxiety (Kasneci et al., 2023).

At the same time, students’ attitudes toward AI are not purely negative. Research suggests that individuals may simultaneously hold appreciation and aversion toward algorithms, recognizing their functional benefits while remaining cautious about their risks (Jin & Li, 2025). In educational settings, the convenience of AI tools and their rapid integration into academic and professional practices may strengthen students’ motivation and perceived need to learn AI. However, the rapid evolution of AI technologies and the growing expectation that students master them may also create additional pressure, even among those who appreciate their value (Kasneci et al., 2023). From this perspective, both algorithm aversion and algorithm appreciation may be relevant to the formation of AI learning anxiety.

Based on the above analysis, this study conceptualizes algorithm appreciation and algorithm aversion as two independent yet coexisting attitudinal dimensions and posits that they jointly influence AI learning anxiety. Accordingly, the following hypotheses are proposed:

**H7.**  *Algorithm aversion has a significant effect on AI learning anxiety.*

**H8.**  *Algorithm appreciation has a significant effect on AI learning anxiety.*

## 4. Methodology

### 4.1. Data Collection and Sampling Method

Participants were recruited from multiple universities across China, covering undergraduate, master’s, and doctoral levels and a range of disciplines, including science and engineering, humanities and social sciences, and the arts. This diversity in academic level and disciplinary background helped improve the breadth and representativeness of the sample. Participants were invited to complete the questionnaire either online or in person through Wenjuanxing (Questionnaire Star). To ensure that respondents had experience relevant to the research topic, screening questions were placed before the main questionnaire items. Only students who had learned to use AI tools (e.g., ChatGPT and DeepSeek) in higher education and had used them to support learning tasks were included. No monetary incentive was offered for participation.

A total of 450 questionnaires were collected through online and offline channels. Based on the data screening criteria, 41 invalid responses were removed, mainly because of unusually short completion times or poor response quality, such as selecting the same option throughout the questionnaire. The final sample included 409 valid responses, and the descriptive statistics are reported in Table 1. All valid respondents were currently enrolled university students (*n* = 409) who had experience learning to use AI tools in educational task contexts and had used such tools at least once per week in the recent past.

The minimum required sample size was estimated using G*Power 3.1.9.7. Assuming *f*^2^ = 0.15, *α* = 0.05, statistical power (1 − *β*) = 0.80, and three predictor variables, the required minimum sample size was 77. The actual sample size of 409, therefore, met this requirement.

### 4.2. Measurement Instrument

The questionnaire items were adapted from previously validated scales and prior studies, with minor modifications made to fit the research context and local language use. All items were measured on a five-point Likert scale (1 = strongly disagree, 5 = strongly agree).

The items measuring the dependent variable, AI learning anxiety, were adapted from the AI anxiety scales used by Y. Y. Wang and Wang (2022) and Y.-M. Wang et al. (2024). These scales include dimensions related to anxiety about learning AI and anxiety about AI replacing human work. In this study, only the items related to AI learning anxiety were selected and adapted to measure the level of anxiety students experienced during the learning process. The items for algorithm aversion were adapted from a scale developed by Jain et al. (2025) to measure employees’ aversion to algorithms and were revised to fit the educational context of the present study. The items for algorithm appreciation were adapted from AI-related emotion and attitude scales developed by Xie et al. (2025) and Choung et al. (2023). Among the antecedent variables, the items for perceived AI explainability were adapted from Shin (2021) and Liu et al. (2022), the items for perceived AI ethical risk were adapted from Uludağ et al. (2025), and the items for AI performance expectancy were adapted from Y. Zhang et al. (2025) and Duong (2024).

Since the original scales were developed in different research contexts, all items were translated and adapted before data collection to fit the context of Chinese university students and AI-supported learning. The translated items were back-translated and compared with the original scales to ensure semantic consistency. Three experts independently reviewed the adapted items to evaluate their relevance and appropriateness for the research questions. After data collection, a preliminary assessment showed acceptable sampling adequacy and internal consistency for the overall questionnaire, with a KMO value of 0.858 and a Cronbach’s alpha of 0.845. Finally, as reported in Section 5.1, the psychometric properties of the adapted scales were further evaluated through reliability, convergent validity, and discriminant validity tests. The specific questionnaire items and their adapted sources are provided in Appendix A.

### 4.3. Data Analysis

This study used a two-stage SEM–ANN approach that combined partial least squares structural equation modeling (PLS-SEM) and artificial neural networks (ANN). This combined approach allowed the study to test the proposed hypotheses and identify key predictors at the same time. In the first stage, SmartPLS 4.1.0.9 was used to estimate the PLS-SEM model and test the hypotheses (Ringle et al., 2024). Because this study aimed to explore the mechanisms and path relationships among variables and was more exploratory than confirmatory, PLS-SEM was chosen instead of covariance-based structural equation modeling (CB-SEM). Compared with CB-SEM, PLS-SEM places greater emphasis on prediction and the maximization of explained variance. It also usually provides higher statistical power and requires fewer assumptions about data distribution, which makes it more suitable for data that deviates from normality and for relatively complex models (Hair et al., 2019). It was therefore appropriate for the objectives and data characteristics of this study. In the second stage, three ANN models were constructed according to the three endogenous constructs in the research model. Ten-fold cross-validation was then applied, and sensitivity analysis was used to calculate the normalized importance of each predictor.

The two methods were combined because structural equation modeling (SEM) and artificial neural networks (ANN) provide different but complementary types of evidence. PLS-SEM is suitable for testing theoretically specified paths, estimating the direction and strength of relationships, and assessing whether the proposed hypotheses are statistically supported. However, when used alone, PLS-SEM mainly evaluates the relationships specified in the structural model and represents these effects through linear path coefficients (Parhi et al., 2022). In behavioral research, students’ psychological responses to artificial intelligence may be influenced by complex combinations of perceived value, perceived risk, and algorithmic attitudes, and may not always be fully captured by a linear structural model. Therefore, ANN was introduced in the second stage as a complementary predictive tool because it does not rely on the same linear specification, can accommodate more flexible relationships among predictors, and enables the assessment of the relative predictive importance of key variables (Leong et al., 2025). Because ANN has a “black-box” nature and is not designed for strict hypothesis testing or causal inference, it was used only as a predictive and complementary method. From a practical perspective, this hybrid approach provides complementary predictive evidence on whether the theoretically specified predictors also show predictive importance when examined through a more flexible modeling method. Accordingly, this study used PLS-SEM to test the theoretical paths and their significance, while using ANN to strengthen the predictive analysis and rank the relative importance of key drivers (Priyadarshinee et al., 2017). By combining these two methods, this study maintained theoretical interpretability while strengthening the identification of key factors associated with AI learning anxiety.

## 5. Results

### 5.1. Measurement Model Assessment

This study assessed internal consistency reliability using Dijkstra–Henseler’s rho_A and composite reliability (CR). As shown in Table 2, all CR values ranged from 0.754 to 0.891, exceeding the recommended threshold of 0.70 and indicating satisfactory internal consistency (Bawack et al., 2021). For rho_A, all constructs except AE (0.636) and AAV (0.695) exceeded 0.70; given the exploratory nature of this study, values between 0.60 and 0.70 were considered acceptable (Dolinting & Pang, 2022). Overall, the measurement model showed adequate reliability for subsequent PLS-SEM analysis.

Convergent validity was evaluated using factor loadings (FL) and average variance extracted (AVE). As reported in Table 2, all loadings were significant (*p* < 0.001). Although AE3, AAP3, and AAP4 were below 0.70, they remained above 0.40, and both AE and AAP met the recommended AVE and CR thresholds. According to Hair et al. (2021), such items may be retained when AVE exceeds 0.50 and CR exceeds 0.70. The AVE values for all constructs ranged from 0.506 to 0.804, all above the minimum threshold of 0.50 (Ab Hamid et al., 2017), indicating adequate convergent validity.

Discriminant validity was assessed using the heterotrait–monotrait ratio (HTMT). As shown in Table 3, all HTMT values were below 0.85, indicating satisfactory discriminant validity for the overall model (Voorhees et al., 2016).

### 5.2. Structural Model Assessment

Overall model fit was assessed using the standardized root mean square residual (SRMR). The SRMR values were 0.080 for the saturated model and 0.094 for the estimated model, both below the commonly used threshold of 0.10, indicating acceptable model fit (Ximénez et al., 2022). Multicollinearity was examined using the variance inflation factor (VIF), and all VIF values ranged from 1.071 to 2.110, well below the critical threshold of 5.00 (Lew et al., 2020). The hypothesized paths were then tested using a bias-corrected and accelerated bootstrap (BCa bootstrap) procedure with 5000 resamples.

As shown in Table 4, APE had significant positive effects on both AAV (*β* = 0.101, *p* = 0.018) and AAP (*β* = 0.315, *p* < 0.001). AE also positively affected AAV (*β* = 0.114, *p* = 0.027) and AAP (*β* = 0.228, *p* < 0.001). AER had a significant positive effect on AAV (*β* = 0.592, *p* < 0.001), but its effect on AAP was not significant (*β* = −0.016, *p* = 0.741). At the outcome level, both AAV (*β* = 0.576, *p* < 0.001) and AAP (*β* = 0.156, *p* < 0.001) positively affected ALA. Thus, all hypotheses were supported except H6.

Effect sizes are also reported in Table 4. APE showed a small effect on AAV (*f*^2^ = 0.014) and a stronger effect on AAP (*f*^2^ = 0.100). AE had small effects on both AAV (*f*^2^ = 0.017) and AAP (*f*^2^ = 0.052). By contrast, AER showed the largest effect on AAV (*f*^2^ = 0.585), while its effect on AAP was negligible (*f*^2^ = 0.000). For ALA, AAV had a large effect size (*f*^2^ = 0.525), whereas AAP had a small effect (*f*^2^ = 0.038). Overall, the pathway AER → AAV → ALA emerged as the most prominent mechanism in the model.

### 5.3. Mediation Analysis

This study further examined the mediating effects of APE, AE, and AER on ALA through AAV and AAP. The mediation effects were estimated using bootstrapping with 5000 subsamples, and the results are presented in Table 5. Overall, all indirect effects were significant except AER → AAP → ALA, and the corresponding bias-corrected confidence intervals did not include zero.

Among all indirect paths, AER showed the strongest indirect effect on ALA through AAV (indirect effect = 0.341, *t* = 11.314, *p* < 0.001), indicating that perceived ethical risk mainly increased AI learning anxiety by strengthening algorithm aversion. By contrast, the indirect effect of AER on ALA through AAP was not significant (indirect effect = −0.003, *t* = 0.318, *p* = 0.751), suggesting that perceived ethical risk did not indirectly affect anxiety through algorithm appreciation.

Both AE and APE showed significant indirect effects on ALA through AAV and AAP. Specifically, AE influenced ALA through AAV (indirect effect = 0.066, *t* = 2.203, *p* = 0.028) and AAP (indirect effect = 0.035, *t* = 2.815, *p* = 0.005). Similarly, APE influenced ALA through AAV (indirect effect = 0.058, *t* = 2.301, *p* = 0.021) and AAP (indirect effect = 0.049, *t* = 2.905, *p* = 0.004). These findings support the proposed dual-path mechanism and again identify AER → AAV → ALA as the dominant indirect pathway.

### 5.4. Predictive Relevance and Explanatory Power

This study used *R*^2^ and *Q*^2^ to assess the explanatory power and predictive relevance of the structural model. The model explained 40.9% of the variance in AAV (*R*^2^ = 0.409), 21.5% of the variance in AAP (*R*^2^ = 0.215), and 37.9% of the variance in ALA (*R*^2^ = 0.379). As shown in Table 6, the *Q*^2^ values for AAV, AAP, and ALA were 0.251, 0.102, and 0.249, respectively, all greater than zero, indicating acceptable predictive relevance for all endogenous constructs (Muzafar et al., 2023). Taken together, these results suggest that the model had satisfactory explanatory power and predictive relevance.

### 5.5. Artificial Neural Network Analysis

**Figure 2:**
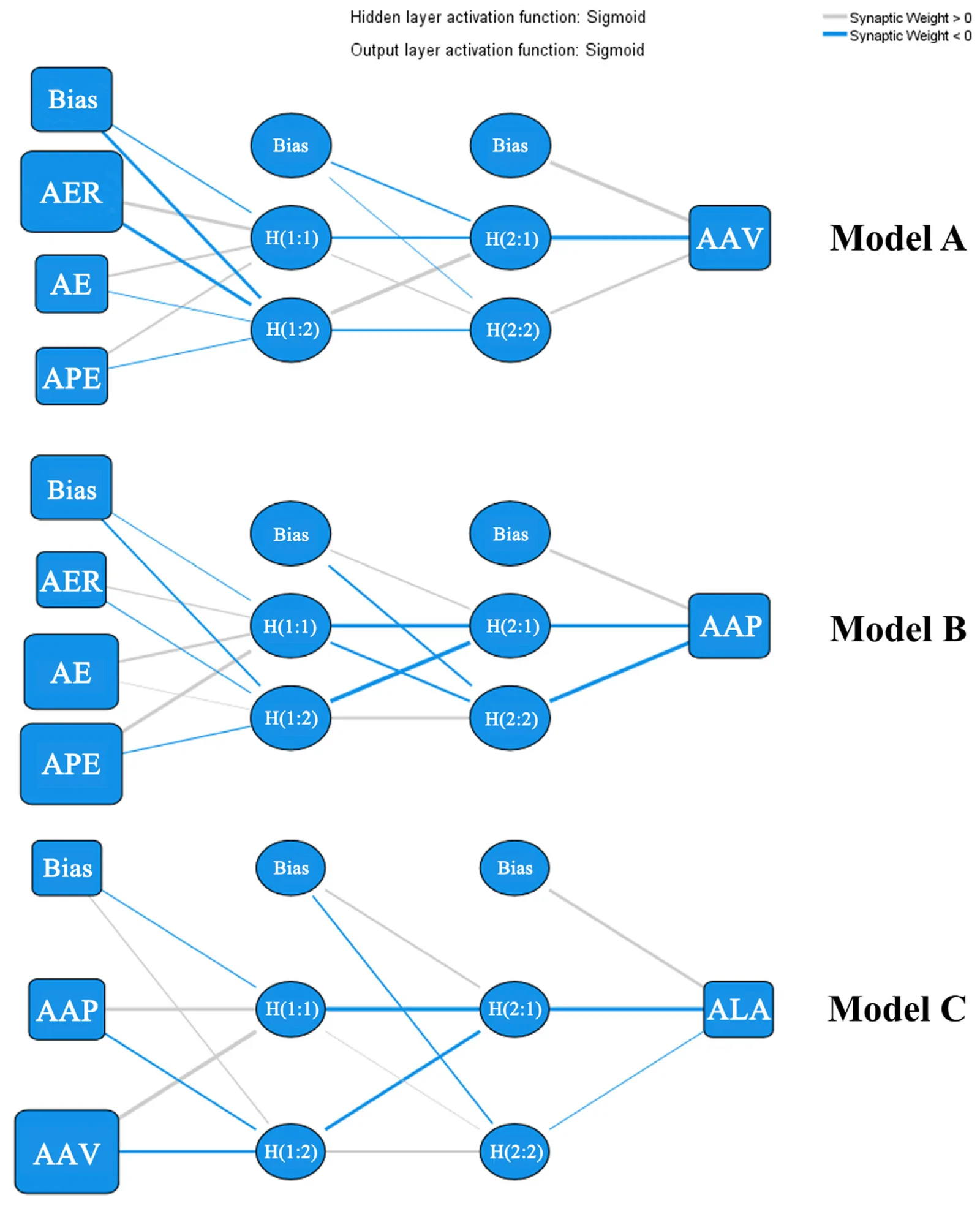
Examples of ANN Model Structures.

Because PLS-SEM mainly captures compensatory and linear relationships, this study additionally employed artificial neural networks (ANN) to explore potential nonlinear relationships and complement the SEM findings. Based on the SEM results, three ANN models were developed for AAV, AAP, and ALA, respectively, as shown in Figure 2. Following Leong et al. (2025), this study adopted a feedforward backpropagation multilayer perceptron ANN with an input layer, hidden layers, and an output layer. A two-hidden-layer structure was used to improve predictive performance, and the feedforward backpropagation (FFBP) algorithm with sigmoid activation functions was applied in both the hidden and output layers. The model was trained using gradient descent optimization.

To reduce overfitting, the ANN models were evaluated using 10-fold cross-validation, with 90% of the sample used for training and 10% for testing in each round. Table 7 presents the root mean square error (RMSE) results. Across the 10 runs, training and testing RMSE values were generally low and stable, indicating satisfactory predictive performance (Sternad Zabukovšek et al., 2019). Mean training and testing RMSE values were 0.111 and 0.098 for Model A, 0.138 and 0.136 for Model B, and 0.123 and 0.121 for Model C.

Table 8 reports the sensitivity analysis (SA) results. For AAV, AER (SA = 100.000%) was the most important predictor, followed by APE (SA = 28.360%) and AE (SA = 27.110%). For AAP, APE (SA = 99.580%) ranked highest, followed by AE (SA = 83.670%) and AER (SA = 22.620%). For ALA, AAV (SA = 100.000%) was the strongest predictor, followed by AAP (SA = 34.990%). Overall, the ANN results were consistent with the SEM findings, again identifying AER, APE, and AAV as the most important predictors of AAV, AAP, and ALA, respectively.

## 6. Discussion

### 6.1. Key Findings

#### 6.1.1. Perceived Ethical Risk Is the Strongest Driver of Algorithm Aversion and AI Learning Anxiety

This study shows that perceived AI ethical risk is the strongest antecedent of algorithm aversion (*β* = 0.592), exceeding the effects of performance expectancy and perceived explainability. This suggests that, in higher education, students’ negative attitudes toward AI are shaped less by whether AI is powerful or understandable and more by concerns about its ethical consequences and potential threats (W. Zhu et al., 2025). Prior studies similarly indicate that AI use may evoke concerns about privacy leakage, data misuse, bias, unfairness, and hallucinated outputs, all of which are especially salient in academically demanding settings where responsibility and academic integrity matter (Ferhataj et al., 2025). This ethical-risk mechanism may be particularly salient in higher education because students’ use of AI is closely connected to coursework, assessment, and academic writing. As the boundaries of acceptable AI use are still being clarified, students may become more sensitive to whether AI-assisted work can be considered original, academically legitimate, and responsibly produced (Lund et al., 2025). This may help explain why perceived ethical risk emerged as the strongest antecedent of algorithm aversion in this study.

At the same time, the path from perceived ethical risk to algorithm appreciation was negative but not significant (*β* = −0.016), suggesting that ethical risk does not simply weaken students’ recognition of AI’s functional value. Rather, students may simultaneously acknowledge AI as useful while remaining cautious or resistant to its ethical risks (Rahim et al., 2022). Given the strong effect of algorithm aversion on AI learning anxiety (*β* = 0.576), perceived ethical risk appears to increase anxiety primarily by strengthening avoidance-oriented attitudes toward AI (H. Wang & Qin, 2025). This finding highlights the central role of ethical threat perception in the formation of AI learning anxiety and suggests that anxiety reduction in educational contexts cannot rely on performance enhancement or explainability alone (J. J. H. Kim et al., 2025).

#### 6.1.2. In Educational Contexts, Explainability May Shift from a Source of Control to a Source of Pressure

The study found that perceived explainability positively predicted algorithm appreciation (*β* = 0.228), which is consistent with prior research showing that clearer and more understandable AI systems are more likely to be trusted and positively evaluated (Shin, 2021). In higher education, explainability may reduce unfamiliarity with AI and strengthen students’ understanding and acceptance of it as a learning support tool (D. Wang et al., 2024).

However, perceived explainability also showed a weaker but significant positive effect on algorithm aversion (*β* = 0.114). This pattern differs from studies that have treated explainability mainly as a resource for reducing uncertainty and resistance (Xu & Wang, 2024). One plausible explanation is that, in educational settings, explainability not only clarifies how AI works but also makes error sources, bias mechanisms, and responsibility attribution more visible (Memarian & Doleck, 2023). As a result, transparency may function not only as a source of control but also as a source of pressure by heightening the salience of risk and accountability (Hassija et al., 2024). These findings suggest that explainability in education is context-dependent: it mainly promotes appreciation, but it may also trigger aversion when it amplifies responsibility awareness.

#### 6.1.3. Performance Expectancy Has a Double-Edged Effect on Algorithm Attitudes

AI performance expectancy significantly increased algorithm appreciation (*β* = 0.315) and also had a weaker positive effect on algorithm aversion (*β* = 0.101), suggesting a double-edged role of expected AI performance (J. Chen et al., 2023). On the one hand, when students believe that AI can improve learning efficiency and task performance, they are more likely to recognize its functional value and develop stronger algorithm appreciation (S. Wang et al., 2023). On the other hand, high performance expectations may also create pressure and a sense of technological threat, especially when students worry that failure to master AI will place them at a disadvantage in academic competition (Hannan & Liu, 2023).

In addition, stronger expectations of AI performance may heighten students’ sensitivity to technological dependence, error consequences, and reduced autonomy (Zhai et al., 2024). Performance expectancy, therefore, appears to promote positive evaluations of AI while simultaneously embedding caution and pressure. When AI is perceived as highly capable, students may not only recognize its usefulness but also become more aware of the extent to which learning tasks could become dependent on algorithmic support (Jia & Tu, 2024). Thus, performance expectancy increases the perceived value of AI while also raising the perceived stakes of using it.

#### 6.1.4. Both Algorithm Appreciation and Algorithm Aversion Can Increase AI Learning Anxiety Through a Dual-Path Mechanism

Our study found that both algorithm aversion and algorithm appreciation significantly increased AI learning anxiety, although the effect of algorithm aversion was much stronger (*β* = 0.576) than that of algorithm appreciation (*β* = 0.156). This indicates that AI learning anxiety is not driven solely by negative evaluations of AI, but may emerge through two parallel psychological paths.

The first is an avoidance path centered on threat appraisal and low perceived control (Stapinski et al., 2010). Students who hold aversive attitudes toward algorithms are more likely to view AI as opaque, unreliable, and potentially harmful in learning contexts, leading to concerns about bias, unstable outputs, academic integrity, and personal inadequacy (Oomen et al., 2024). Because negative information typically carries greater psychological weight, such concerns are especially likely to translate into anxiety (Rozin & Royzman, 2001).

The second is a pressure path characterized by high engagement and high expectations. Students who appreciate AI may use it more frequently and rely on it more heavily, which exposes them more directly to pressures related to prompt use, result verification, rapid tool iteration, and social or ability comparison (Li & Jiang, 2025). In this sense, positive attitudes may also generate anxiety by increasing the perceived necessity of learning AI well (Frenkenberg & Hochman, 2025).

These findings can be understood through the lens of approach–avoidance conflict (Elliot, 2006). Students may be drawn to AI because of its value and utility while simultaneously resisting it because of its uncertainty and risks. The coexistence of these two attitudes creates sustained psychological tension, thereby increasing AI learning anxiety. Among the two paths, the threat-based pathway appears more direct and psychologically potent, which helps explain why algorithm aversion exerts the stronger effect.

These mechanisms may be particularly salient in the context of higher education in China. Students in this context often face strong performance pressure related to academic credentials, postgraduate admission opportunities, and future employment (Zhuang & Liu, 2025). As AI becomes increasingly embedded in learning and academic work, it may be perceived not only as a learning tool but also as an emerging competence and productivity resource (Fošner, 2024). This adoption climate may intensify perceived technological competition and amplify the pressure pathway identified in this study: even students who appreciate AI’s value may feel the need to keep pace with rapidly evolving tools in order to maintain their academic and professional competitiveness (Hannan & Liu, 2023). These findings suggest that AI learning anxiety is shaped not only by individual algorithm attitudes but also by the institutional and cultural conditions under which AI is adopted in higher education.

### 6.2. Theoretical and Practical Implications

This study contributes to the literature by showing that students’ attitudes toward AI in higher education are not adequately captured by a simple positive-negative distinction. Algorithm appreciation and algorithm aversion can exist at the same time, and both are associated with AI learning anxiety. This means that valuing AI does not necessarily make the learning process feel easier or more secure. Instead, anxiety appears to arise when students see AI as useful, yet remain uncertain about its reliability, limits, and consequences (Hornbæk & Hertzum, 2017).

The findings also refine current understanding of explainability and ethical risk in educational settings. Explainability is usually discussed as a factor that improves trust, but in this study, it also showed a positive link with algorithm aversion. In higher education, clearer explanations may help students understand AI, while also making them more aware of possible errors and of their own responsibility for judging AI outputs. Ethical risk plays an even more central role. The results suggest that students’ anxiety is shaped not only by what AI can do, but by whether its use feels legitimate, safe, and governable within academic work (B. Zhang et al., 2025).

The practical implication is that universities should not treat AI learning anxiety as a problem that can be solved through access or training alone. Students need a clearer framework for using AI in ways they can justify and manage. In the Chinese higher education context, such support is especially important because AI adoption may become closely linked to academic competitiveness, future employability, and students’ perceived need to keep pace with their peers (Yan et al., 2025). Institutions should therefore give more explicit guidance on academic integrity, privacy, verification, and responsibility in AI-assisted learning (Z. Chen et al., 2024). Instruction on AI-supported learning should also help students develop critical judgment about AI outputs, rather than focusing only on operational familiarity with AI tools. Tasks that require checking sources, verifying outputs, and correcting errors may help students build a more stable sense of control when working with AI (Lin & Chen, 2024). From this perspective, effective AI support in education depends not only on system capability, but on whether the learning environment reduces avoidable uncertainty and helps students engage with AI in a more assured way (Geethanjali & Umashankar, 2025).

### 6.3. Limitations

The study also has certain limitations. First, its cross-sectional design identifies associations rather than causal relationships. Dynamic reciprocal effects may exist among AI learning anxiety, algorithm attitudes, and their antecedents. Future longitudinal studies could help address this issue by measuring these variables at multiple time points, thereby establishing temporal ordering and examining whether earlier algorithm attitudes predict later changes in AI learning anxiety. Experimental studies could further strengthen causal inference by manipulating explainability cues, ethical risk information, or AI performance feedback and observing whether these manipulations lead to changes in algorithm appreciation, algorithm aversion, and AI learning anxiety. Second, the sample was drawn mainly from Chinese universities and consisted of students with prior AI use experience, which may limit the generalizability of the findings to other educational and cultural contexts. The relatively high proportion of master’s students also suggests that differences across academic levels warrant further investigation. Third, the study relied primarily on self-report data, which may not fully capture students’ actual reactions and behaviors in real learning settings. Future research could therefore incorporate behavioral data, real learning tasks, classroom observation, and interviews to improve ecological validity and strengthen the explanatory power of the findings.

## 7. Conclusions

In the context of higher education in China, this study examined how AI performance expectancy, perceived explainability, and perceived ethical risk shape students’ algorithm appreciation and algorithm aversion, and how these attitudes are associated with AI learning anxiety. The findings reveal the double-edged role of algorithm attitudes: although algorithm aversion had a stronger effect on AI learning anxiety, algorithm appreciation also showed a significant positive effect, suggesting that positive recognition of AI’s learning value may coexist with anxiety. Perceived ethical risk was the strongest predictor of algorithm aversion and constituted the most prominent indirect pathway to AI learning anxiety. Meanwhile, performance expectancy and perceived explainability enhanced algorithm appreciation while also increasing algorithm aversion to a lesser extent. This suggests that, in educational contexts, usefulness and transparency may simultaneously foster positive attitudes toward algorithms while heightening concerns about risks or accountability. The findings suggest that future AI integration in higher education should be accompanied by clearer ethical guidance, verification practices, and emotionally supportive AI learning environments.

## Figures and Tables

**Table 1:** Sample distribution and descriptive statistics.

	Category	Sample Size	Percentage (%)
Gender	Male	167	40.83
	Female	242	59.17
Current stage of study	Undergraduate Student	116	28.36
	Master’s Student	260	63.57
	Doctoral Student	33	8.07
Field of study	Humanities and Social Sciences	91	22.25
	Science and Engineering	157	38.39
	Arts	161	39.36

**Table 2:** Loadings, composite reliability, Dijkstra Henseler and average variance extracted.

Constructs	Items	Loadings (*p*-Levels)	rho_A	CR	AVE
AI Performance Expectations (APE)	APE1	0.850 (*p* < 0.001)	0.770	0.867	0.684
	APE2	0.824 (*p* < 0.001)			
	APE3	0.808 (*p* < 0.001)			
Perceived AI Explainability (AE)	AE1	0.859 (*p* < 0.001)	0.636	0.754	0.517
	AE2	0.760 (*p* < 0.001)			
	AE3	0.486 (*p* < 0.001)			
Perceived AI Ethical Risks (AER)	AER1	0.921 (*p* < 0.001)	0.786	0.891	0.804
	AER2	0.872 (*p* < 0.001)			
Algorithms Aversion (AAV)	AAV1	0.792 (*p* < 0.001)	0.695	0.831	0.621
	AAV2	0.788 (*p* < 0.001)			
	AAV3	0.783 (*p* < 0.001)			
Algorithms Appreciation (AAP)	AAP1	0.802 (*p* < 0.001)	0.727	0.801	0.506
	AAP2	0.799 (*p* < 0.001)			
	AAP3	0.640 (*p* < 0.001)			
	AAP4	0.575 (*p* < 0.001)			
AI Learning Anxiety (ALA)	ALA1	0.811 (*p* < 0.001)	0.837	0.891	0.672
	ALA2	0.848 (*p* < 0.001)			
	ALA3	0.856 (*p* < 0.001)			
	ALA4	0.760 (*p* < 0.001)			

**Table 3:** Heterotrait–monotrait ratio (HTMT) results.

	ALA	AAP	AAV	AER	AE	APE
AI Learning Anxiety						
Algorithms Appreciation	0.280					
Algorithms Aversion	0.778	0.198				
Perceived AI Ethical Risks	0.423	0.088	0.838			
Perceived AI Explainability	0.470	0.546	0.325	0.149		
Perceived AI Performance Expectations	0.563	0.563	0.295	0.142	0.619	

**Table 4:** Structural model results.

Hypothesis	Path Coefficients	Sample Mean	STDEV	*t*-Value	*p*-Value	*f* ^2^	Remarks
H1: APE → AAV	0.101	0.102	0.043	2.375	0.018	0.014	Supported
H2: APE → AAP	0.315	0.317	0.054	5.856	0.000	0.100	Supported
H3: AE → AAV	0.114	0.116	0.052	2.209	0.027	0.017	Supported
H4: AE → AAP	0.228	0.231	0.054	4.232	0.000	0.052	Supported
H5: AER → AAV	0.592	0.591	0.038	15.651	0.000	0.585	Supported
H6: AER → AAP	−0.016	−0.016	0.049	0.331	0.741	0.000	Not Supported
H7: AAV → ALA	0.576	0.577	0.032	17.998	0.000	0.525	Supported
H8: AAP → ALA	0.156	0.157	0.040	3.905	0.000	0.038	Supported

**Table 5:** Results of the mediation effect test.

	Original Sample	Sample Mean	*t*-Statistics	*p*-Values	BCCI	
					2.5%	97.5%
AER → AAV → ALA	0.341	0.341	11.314	0.000	0.282	0.399
AER → AAP → ALA	−0.003	−0.003	0.318	0.751	−0.019	0.012
AE → AAV → ALA	0.066	0.067	2.203	0.028	0.009	0.126
AE → AAP → ALA	0.035	0.036	2.815	0.005	0.014	0.063
APE → AAV → ALA	0.058	0.059	2.301	0.021	0.011	0.109
APE → AAP → ALA	0.049	0.050	2.905	0.004	0.021	0.087

**Table 6:** Predictive relevance (*Q*^2^) and explanatory power (*R*^2^).

Endogenous Construct	*Q* ^2^	Predictive Relevance	*R* ^2^
Algorithms Aversion	0.251	*Q*^2^ > 0	0.409
Algorithms Appreciation	0.102	*Q*^2^ > 0	0.215
AI Learning Anxiety	0.249	*Q*^2^ > 0	0.379

**Table 7:** RMSE for neural network model.

Neural Network	Model A		Model B		Model C	
	Input: APE, AE, AER		Input: APE, AE, AER		Input: AAV, AAP	
	Output: AAV		Output: AAP		Output: ALA	
	Training	Testing	Training	Testing	Training	Testing
	RMSE	RMSE	RMSE	RMSE	RMSE	RMSE
ANN1	0.112	0.081	0.133	0.130	0.119	0.115
ANN2	0.110	0.110	0.133	0.118	0.120	0.144
ANN3	0.110	0.104	0.137	0.126	0.122	0.121
ANN4	0.113	0.071	0.138	0.130	0.132	0.122
ANN5	0.109	0.096	0.130	0.145	0.123	0.100
ANN6	0.108	0.104	0.139	0.142	0.125	0.114
ANN7	0.110	0.101	0.141	0.134	0.125	0.114
ANN8	0.110	0.108	0.131	0.155	0.121	0.133
ANN9	0.113	0.100	0.135	0.112	0.121	0.116
ANN10	0.111	0.100	0.160	0.168	0.121	0.134
Mean	0.111	0.098	0.138	0.136	0.123	0.121
SD	0.002	0.012	0.009	0.017	0.004	0.013

**Table 8:** ANN results and sensitivity analysis.

Neural Network	Model A (Output: AAV)			Model B (Output: AAP)			Model C (Output: ALA)	
	AER	APE	AE	APE	AE	AER	AAV	AAP
ANN1	0.629	0.180	0.191	0.454	0.437	0.109	0.755	0.245
ANN2	0.714	0.067	0.219	0.486	0.414	0.100	0.754	0.246
ANN3	0.678	0.156	0.166	0.475	0.404	0.121	0.720	0.280
ANN4	0.689	0.128	0.182	0.444	0.459	0.096	0.626	0.374
ANN5	0.656	0.191	0.154	0.536	0.404	0.061	0.675	0.325
ANN6	0.657	0.188	0.156	0.499	0.285	0.216	0.747	0.253
ANN7	0.689	0.166	0.145	0.490	0.407	0.102	0.805	0.195
ANN8	0.644	0.235	0.120	0.486	0.398	0.116	0.819	0.181
ANN9	0.531	0.274	0.195	0.443	0.447	0.110	0.767	0.233
ANN10	0.590	0.200	0.210	0.544	0.395	0.061	0.784	0.216
Average relative importance	0.648	0.179	0.174	0.486	0.405	0.109	0.745	0.255
Normalized relative importance (%)	100.000	28.360	27.110	99.580	83.670	22.620	100.000	34.990

## Data Availability

The data presented in this study are available on request from the corresponding author due to ethical and confidentiality restrictions stated in the participant informed consent (i.e., raw questionnaire data are stored on encrypted devices and accessible only to the research team).

## Abbreviations

The following abbreviations are used in this manuscript:

APE	AI Performance Expectations
AER	AI Ethical Risks
AAP	Algorithm Appreciation
AAV	Algorithm Aversion
ALA	AI Learning Anxiety
AE	AI Explainability

## Appendix A

The questionnaires used in this study and the sources from which they were adapted are listed in Table A1.

**Table A1:** Research Questionnaire.

Variables	Code	Questions	References
AI Performance Expectations	APE1	I find AI useful in my studies.	(Y. Zhang et al., 2025; Duong, 2024)
	APE2	Using AI helps me accomplish learning tasks more effectively.	
	APE3	Using AI increases my productivity in my studies.	
Perceived AI Explainability	AE1	I found that AI algorithms are easily understandable.	(Shin, 2021; Liu et al., 2022)
	AE2	I think AI algorithms are explainable.	
	AE3	I think AI algorithms can provide clear explanations when used to support learning.	
Perceived AI Ethical Risks	AER1	AI may produce unreliable results, which could pose risks to students’ academic integrity.	(Uludağ et al., 2025)
	AER2	I am concerned about the ethical dilemmas associated with using AI tools to support my studies.	
Algorithm Aversion	AAV1	I feel reluctant to interact with and use AI algorithms in learning.	(Jain et al., 2025)
	AAV2	With the increasing use of AI in learning, I think that students’ academic learning experiences may change for the worse.	
	AAV3	I think that AI algorithms may weaken students’ own ways of learning.	
Algorithm Appreciation	AAP1	I appreciate the value of algorithmic support in my learning.	(Xie et al., 2025; Choung et al., 2023)
	AAP2	I feel positive about using algorithm-generated suggestions or feedback in my studies.	
	AAP3	I think using AI tools to support learning is a good idea.	
	AAP4	I regard algorithm-supported learning as a smart way to handle learning tasks.	
AI Learning Anxiety	ALA1	Learning to understand the functions of AI tools makes me anxious.	(Y. Y. Wang & Wang, 2022; Y.-M. Wang et al., 2024)
	ALA2	Learning to use AI tools to support my studies makes me anxious.	
	ALA3	Learning how AI tools work makes me anxious.	
	ALA4	Being unable to keep up with advances in AI tools makes me anxious.	
